# Supplementary material for: Application of Novel Technologies in Cardiac Electrotherapy to Prevent Complications
Source: Diagnostics (Basel). 2023 Apr 28;13(9):1584. doi: 10.3390/diagnostics13091584 (PMC10178181; doi:10.3390/diagnostics13091584)
Supplement: Supplementary file 1 [file diagnostics-13-01584-s001.zip › Supplementary Table S1.pdf]

Supplementary Table S1. Summary of studies reporting lead dislodgment into the pulmonary artery.

Author,	Country, year	Studied population	Main findings	Treatment	Efficacy
**Original research**					
Polewczyk, M.; et al. [13]	Poland, 2016	1767 TLEs was carried out, including a group of 19 patients with LDPA	The presence of LDPA was associated with frequent occurrence of intracardiac lead abrasion (increase by 316%) and isolated LRIE (increase by 500%)	TLE	Long-term mortality after TLE was comparable in patients with LDPA are comparable to those in standard TLE procedures
Paskudzka, D.; et al. [14]	Poland, 2018	3 patients (patient A 63 year old woman, patient B 31 year old woman, patient C 83 yea old woman)	Patient A: mild PR and TR in TTE<br><br>Patient B: no signs, dislocation seen on the CXR<br><br>Patient C:<br>- clinically: symptomatic bradycardia 35/min, dizziness, fatigue,<br>- ECG: periodic ineffective ventricular pacing<br>- TTE: PR<br>- device control: increased capture threshold and impedance of the ventricular lead	Patient A: conservative treatment, Patient B: reduction of the lead body excess during the pulse generator replacement, Patient C: TLE and a new CIED implantation	Patient A: stable lead position, stable electrical and TTE parameters. Patient B : a proper lead position. Patient C: systematically increasing TR, features of PH with a proper position of the implanted lead in control TTE
**Case reports**					
Dalvi, B.V.; et al. [26]	India, 1990	Not precised, adult	pacemaker lead migration to the RPA with septic pulmonary embolism	Cefuroxamine i.v.	Not precised
Drögemuller, A.; et al. [23]	Germany, 2003	Adult patient	Split pacemaker lead in the PA, hemorrhagic pleural effusion, increase in stimulation treashold	Catheter-based rescue of displaced lead, capping and fixing	Good clinical effect
Golzio, P.G.; et al. [29]	Italy, 2007	Not precised, adult	CXR: inner coil of an atrial lead in the RPA	TLE	Not precised
Udyavar, A.R.; et al. [22]	India, 2008	56-year-old man	Repeated lead fracture and migration of the lead into pulmonary circulation	Pacemaker implantation with a tined steroid eluting	Not precised

			Clinically: recurrent syncope<br><br>Device control: battery 0%<br><br>CXR: migration of the lead into pulmonary circulation	lead; due to complicated anatomy (azygous vein draining into SVC) no extraction of the earlier endocardial leads was made	
Tay, J.K.Y.; et al. [17]	Edinburgh, 2009	74-year-old man	Device control: increased capture threshold,<br><br>CXR: linear shadow in the left lung, fracture of the lead into 3 segments, one of them migrated into the LPA and the lung, loop of electrode in RV and atrium	Lead extraction and implantation of new pacemaker, a 15 cm loose fragment of lead was removed from RA	repeated CXR suggested further migration of the lead fragment within the PA
Małecka, B.; et al. [32]	Poland, 2010	27-year-old woman	Irregular electric discharges and noises made by ICD, displacement of the left lead end to RPA	Removal of the displaced lead, implantation of new ICD	Discharged home in good general condition
Stein, A.; et al. [28]	Germany, 2011	49-year-old patient	Recurrent VF and VT caused by pacemaker lead migrated to the pulmonary trunk	Not precised	Not precised
Erkan, H.; et al. [18]	Turkey, 2014	78-year-old man	In TTE and CT right abandoned subclavian lead had migrated to the PA. Proximal tip was embolisated	No intervetion	No complications and no clinical signs, the lead remained silent
Polewczyk, M.; et al. [16]	Poland, 2015	56-year-old man	Clinically: signs of LRIE, shortness of breath, fever, cough<br><br>Chest CT: signs of inflammation of mid-right pulmonary lobe, migration of the lead to the PA and lungs<br><br>TTE: dilatation of RA and RV, high PASP (80 mHg)<br><br>TEE: no vegetations	TLE	No complications, no signs of lead fragments or vegetations in TTE/TEE, reduction of PASP (60 mmHg)

Michalak, M.; et al. [15]	Poland, 2015	57-year-old man	Complete breakdown and migration of the lead into PA<br><br>Clinically: mild asymptomatic bradycardia, pectoral muscle contractions depending on body position,<br><br>pacemaker control: lead impedance >2000 Ohms, lack of proper sensing and stimulation<br><br>CXR: lead breakage at the level the right clavicle, migration of the proximal fragment of the broken lead to the LPA	TLE	Patient was discharged home 3 days after the procedure in good general condition
Enab, H.; et al. [25]	England, 2021	89-year-old man	Device control: RV threshold rise<br><br>CXR: first - loop of RV lead in the RVOT, later on displacement of the lead to RPA	Extraction of the lead and implantation of the new RV lead	No complications
Kumar, R.; et al. [24]	India, 2022	8-year-old boy	Clinically: fever, cyanosis, clubbing<br><br>TTE: atrial vegetations and moderate TR<br><br>CXR: prolapsed lead loop into the RVOT and pulmonary valve<br><br>ECG: VT	Antibiotics, emergency surgery - closure of ASD, removal of pulse generator and endocardial leads, pulmonary valve repair	Mild TR, resolution of pulmonary stenosis

Abbreviations: ASD atrial septal defect; CIED cardiac implantable electronic device; CT computed tomography; CXR chest X-ray; ECG electrocardiography; ICD implantable cardioverter-defibrillator; LDPA leads dislodged into the pulmonary artery; LPA left pulmonary artery, LRIE lead-related infective endocarditis; PA pulmonary artery; PASP pulmonary artery systolic pressure; PH pulmonary hypertension; PR pulmonary regurgitation; RA right atrium, RPA right pulmonary artery; RV right ventricle, RVOT right ventricle outflow tract; SVC superior vena cava; TEE transesophageal echocardiography; TLE transvenous lead extraction; TR tricuspid regurgitation; TTE transthoracic echocardiography; VF ventricular fibrillation; VT ventricular tachycardia
